# Supporting Parents of Adolescents With Intellectual Disabilities: A Systematic Review of Interventions

**DOI:** 10.1111/jar.70004

**Published:** 2025-02-19

**Authors:** Emma Scripps, Daniel Sutherland, Peter E. Langdon, Richard P. Hastings, Kylie M. Gray

**Affiliations:** ^1^ Centre for Research in Intellectual and Developmental Disabilities (CIDD) University of Warwick Coventry UK; ^2^ Intellectual Disabilities Research Institute (IDRIS) University of Birmingham Birmingham UK; ^3^ Herefordshire and Worcestershire Health and Care NHS Trust Worcester UK; ^4^ Birmingham Community Healthcare NHS Foundation Trust Birmingham UK; ^5^ Department of Psychiatry, School of Clinical Sciences at Monash Health Monash University Clayton Australia

**Keywords:** adolescents, intellectual disability, interventions, parenting, parents

## Abstract

**Background:**

This review aimed to synthesise the literature about interventions for parents of adolescents with intellectual disability, including parental experience of receiving interventions and intervention effectiveness.

**Methods:**

Eligible interventions aimed to improve parenting skills and/or parent–adolescent relationships, adolescent behavioural/emotional problems and/or parent well‐being. ASSIA, EMBASE, Medline, PsycINFO and Web of Science were last searched on 11 July 2024. The TIDieR checklist and the MMAT were used for quality appraisal. A narrative synthesis was conducted. (Pre‐registration: PROSPERO CRD42022384409).

**Results:**

Twelve studies with 1041 families were included. Intervention descriptions were detailed; however, study quality was mixed. Nearly all studies reported the intervention was associated with positive effects on parenting, parent–adolescent relationships, adolescent behaviour/emotional problems or parent well‐being. Three randomised controlled trials (RCTs) offer the strongest evidence but are limited in quality. All studies investigating parent experiences reported positive responses.

**Conclusions:**

High‐quality studies (e.g., RCTs) are needed to enable conclusions about efficacy and effectiveness.

## 1 Introduction

Adolescence is an important developmental period, with marked physical, social, emotional and cognitive changes, all of which contribute to mental health vulnerability. The onset and prevalence of mental health problems peak during adolescence and early adulthood (Kessler et al. 2007). Adolescents with intellectual disability are particularly at risk of mental health problems and have been found to exhibit more behavioural and mental health problems relative to their peers (Bailey et al. 2019; Einfeld, Ellis, and Emerson 2011). Parents of adolescents with intellectual disability, in comparison to parents of typically developing adolescents, are also at risk for increased mental health problems and reduced well‐being. For example, in a cross‐sectional comparison study in Australia, parents of adolescents with intellectual disability reported experiencing more parental stress than caregivers of typically developing adolescents (Patton et al. 2018). Furthermore, Lin et al. (2009) found that caregivers of children and adolescents with intellectual disability in Taiwan reported lower levels of psychological well‐being than the general population.

Given increased behavioural and mental health problems in adolescents with intellectual disability and the reduced well‐being of their parents, there is a need for effective interventions. A recent systematic review and meta‐analysis aimed to determine the effectiveness of psychological therapy for mental health problems amongst individuals with intellectual disability and three studies where the intervention was delivered directly to children or adolescents were included (Tapp et al. 2023). However, adolescent behavioural and mental health problems and parent well‐being may also be addressed via interventions working directly with or through parents. Such interventions may include parenting/parent training (e.g., Mazzucchelli, Jenkins, and Sofronoff 2018), mental health/well‐being interventions targeting parents (e.g., Flynn et al. 2020) and interventions that work primarily through parents to improve adolescent outcomes (as opposed to direct interventions with adolescents only) (e.g., de Bruin et al. 2015).

Previous systematic reviews and meta‐analyses conducted on parenting, parent well‐being or interventions working through parents to influence adolescent outcomes have focused on parents of children with developmental disabilities (Hohlfeld, Harty, and Engel 2018; Singer, Ethridge, and Aldana 2007), children with traumatic brain injury (Brown et al. 2013), autistic children and adolescents (Conrad et al. 2021), older autistic children and adults (Rutherford et al. 2019), typically developing adolescents with behavioural problems (Medlow et al. 2016) and typically developing adolescents engaging in risk behaviours (Champion et al. 2022; Ladis et al. 2019). However, a review has yet to examine interventions for parents of adolescents with intellectual disability.

The current systematic review aimed to synthesise the existing evidence about parenting interventions, parent well‐being interventions and interventions working through parents to influence adolescent outcomes.

We addressed the following review questions:

1) What parenting interventions, parent well‐being interventions and interventions working through parents to influence adolescent outcomes have been described in the literature for parents of adolescents with intellectual disability?

2) What is the evidence for the effectiveness of these interventions in improving (a) parenting skills and/or the parent– adolescent relationship; (b) adolescent behavioural and emotional/mental health problems or (c) parental mental health and well‐being?

3) What are the experiences of parents receiving these interventions?

## 2 Methods

The protocol for this systematic review was prospectively registered on PROSPERO (CRD42022384409) and this report follows PRISMA reporting guidelines (Page et al. 2021) (see Supplementary Information S1 and Supplementary Information S2 for PRISMA checklists).

### 2.1 Eligibility Criteria

#### 2.1.1 Population

To be eligible, study participants had to be the primary caregiver/s (including biological, adoptive, foster or stepmothers or fathers or primary guardians) of an adolescent with an intellectual disability. This could be one or more parental caregivers from the same family. Whether an adolescent had an intellectual disability could be confirmed by a family member, evidence of receiving intellectual disability education or services, meeting a diagnostic threshold, including a borderline level of general intellectual functioning (Full Scale IQ ≤ 75), or a genetic syndrome strongly associated with intellectual disability. Adolescence was defined as between 10 and 19 years (World Health Organisation 2022). Studies reporting evidence from a group in which ≥ 75% of adolescents met the intellectual disability criterion and ≥ 75% also met the age criterion were eligible. Studies of adolescents with a specific learning disability such as dyslexia or other neurodevelopmental conditions (in the absence of intellectual disability) were not eligible.

#### 2.1.2 Interventions

Studies were eligible for inclusion if they involved parenting, parent well‐being or an intervention working through parents to influence adolescent outcomes. Interventions that had an adolescent component in addition to the parent component were eligible. Interventions delivered directly and only to the adolescent with an intellectual disability, or primarily focused on improving the adolescent's educational achievement (e.g., reading interventions), physical health (e.g., weight loss interventions), the understanding of intellectual disability diagnosis or family relationships other than parent–adolescent (e.g., spousal or sibling relationship) were excluded. All contexts of intervention delivery were eligible including face‐to‐face, online, self‐delivery with telephone or online guidance, or within the home, education, health or community‐based contexts.

#### 2.1.3 Comparator

Studies with or without a control group or comparison sample were eligible.

#### 2.1.4 Outcomes

Studies were eligible if they reported one of the following outcomes:

1. Parenting; including measures of parenting style, parenting self‐efficacy, confidence or competence and/or the parent–adolescent relationship such as measures of closeness or relationship quality.
2. Adolescent behavioural and/or emotional/mental health problems, including conduct problems, peer relationships, prosocial behaviour or externalising/internalising problems.
3. Parent mental health/well‐being such as measures of depression, anxiety, stress or life satisfaction.

For review question three, the outcomes of interest were experiences of parents participating in interventions. Parent experience was defined as any feedback parents provided regarding their participation in the intervention, including satisfaction, reports of relevance or helpfulness and perceptions of impact. Studies including any data (qualitative or quantitative) considering parental experiences of these interventions were eligible.

#### 2.1.5 Other Eligibility Criteria

All research designs and methods (quantitative and qualitative or mixed methods) were eligible for inclusion. However, articles that did not present primary data such as systematic/narrative reviews and book chapters, studies with fewer than three participants and conference abstracts were not eligible. Articles were excluded if they were not available in English.

### 2.2 Information Sources

Studies were identified through systematic searches of five databases: Applied Social Sciences Index and Abstracts (ASSIA), EMBASE, Medline, PsycINFO and Web of Science. Relevant titles were then subject to abstract and subsequent full‐text screening. Corresponding authors of the included studies were also contacted about potentially relevant unpublished or unidentified studies. Once the abstract and full‐text screening was complete, forward and backward citation searching of the included articles was conducted and a full‐text review was completed for any identified studies. Searches were last conducted before completion of the final synthesis (11 July 2024).

### 2.3 Search Strategy

Search term groups included intellectual disability, adolescents, parents/caregivers and intervention. Terms in each of these groups were separated with ‘OR’ and each group combined with ‘AND’ (see the Supplementary Information S3 for the full search strategies). Searches were not restricted by publication date.

### 2.4 Selection Process

Once the searches were complete, all articles were exported to EndNote and all duplicates and non‐English studies were removed electronically and manually. Articles were then screened according to their titles and abstracts. A second reviewer independently screened 100% of titles and abstracts with substantial agreement (98.4%, Cohen's kappa = 0.65). Disagreements were resolved by discussion between the two reviewers. Screening of full texts was then conducted, recording reasons for exclusion with a second reviewer also screening 100% of articles. A good agreement rate was achieved (97.8%, Cohen's kappa = 0.9) and disagreements between the two reviewers were again resolved through discussion.

### 2.5 Data Extraction

A bespoke data extraction form based on the Cochrane data collection template for intervention reviews (The Cochrane Collaboration 2014) was used to extract data with changes to ensure relevance (e.g., additional categories for participant characteristics). Data extracted included (a) article characteristics (title, author(s), year of publication and country); (b) study characteristics (type, aims/research question(s), sample size, inclusion/exclusion criteria, recruitment method, withdrawals/exclusions); (c) participant characteristics (adolescent and parent age, sex, ethnicity/race, intellectual disability severity, co‐morbidities and any other relevant sociodemographic information); (d) intervention characteristics and (e) study outcomes (outcome type, validity of measures, individual reporting, scales, analysis, results of intervention group and control if present, missing participants and reasons and appropriateness of analysis methods). Any missing information was requested from the authors. If no response was received within 28 days, this information was recorded as not reported. Data were also independently extracted from 100% of articles by the second reviewer, discrepancies were highlighted by the first reviewer and a discussion was held between reviewers to resolve differences. For outcome measures, overall scores or sub‐scales were extracted if present. No limitations were placed on the number of times outcome measures were conducted or the length of follow‐up time.

### 2.6 Quality Assessment

The Template for Intervention Description and Replication (TiDieR) checklist (Hoffmann et al. 2014) was used to assess the quality of intervention reporting. This 12‐item checklist comprises information that is required to present an acceptable description of an intervention, with a total score out of 12. A second reviewer independently completed the TiDieR checklist for all studies and differences were resolved by the reviewers through discussion. The Mixed Methods Appraisal Tool (MMAT, Hong et al., 2018) was used to assess methodological quality by providing questions for all study designs and generating a total MMAT score (Table 9). As this review included studies with a variety of methodologies, this tool was most appropriate. Furthermore, the MMAT has been found to be reliable for study method appraisal (Pace et al. 2012). The MMAT requires assessors to respond with ‘yes,’ ‘no’ or ‘can't tell’. A second reviewer independently appraised 100% of the selected studies for this review and an agreement rate of 77.69% (Cohen's kappa = 0.57) was achieved. Subsequently, all discrepancies were resolved through discussion between the two reviewers. As the MMAT quality criteria differ based on study design, caution is required when using an MMAT total score to compare studies of different methodologies. For mixed methods studies, the overall quality score on the MMAT is determined as the lowest score of the qualitative, quantitative and mixed method components, as a study's overall quality is considered to be equivalent to its weakest component (Pace et al. 2012).

### 2.7 Data Synthesis

A narrative synthesis was conducted based on the range of study designs and methods used. A meta‐analysis was deemed inappropriate because study design and quality were diverse. Interventions were grouped into parenting, parent well‐being and interventions working through parents to influence adolescent outcomes. To address review question one, the TIDieR checklist was used (Hoffmann et al. 2014) as summarised in Tables 1, 3 and 6, to both extract relevant intervention detail and to evaluate the quality of intervention reporting overall. For review question two, a summary of the effectiveness of interventions is displayed in Tables 2, 4 and 7. In terms of review question three, a summary of parents' experiences is presented in Tables 5 and 8.

## 3 Results

### 3.1 Study Selection

**FIGURE 1:**
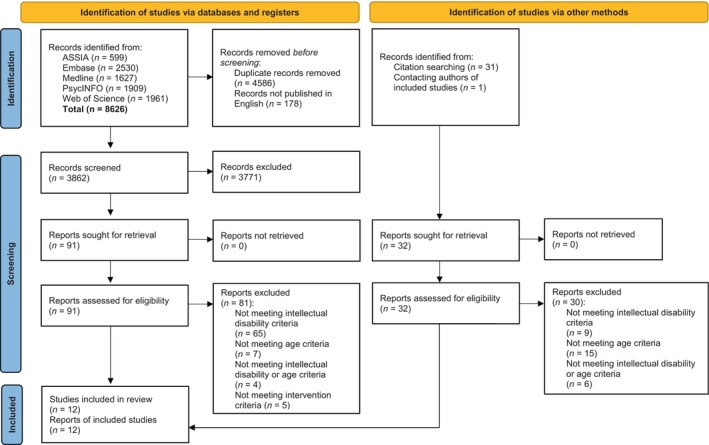
Article identification illustrated using the PRISMA flow diagram (Page et al. 2021).

The PRISMA flow diagram (Page et al. 2021) (Figure 1) illustrates the process of article identification. Searches of the five databases identified 8626 articles, electronic and manual deduplication removed 4586 articles and 178 articles not published in English. Subsequently, 3771 records were excluded at the title and abstract screening based on the previously highlighted eligibility criteria, resulting in 91 articles being subject to full‐text screening. At this stage, 81 articles were excluded as they did not meet the eligibility criteria and 10 articles were determined to be eligible for inclusion. Forward and backward citation searching provided two additional eligible articles. No further eligible studies were derived from contacting authors. Colvin (2013) is an example of a study that appeared to meet the eligibility criteria but was excluded; adolescents met the age and intellectual disability criteria but the family support component of the intervention involved other family members and some families did not utilise the family support.

### 3.2 Study Characteristics

The 12 studies in the review included 1041 families; 634 parents received an intervention themselves, and 407 families received interventions with parent and adolescent components. A total of 863 primary caregivers described themselves as female or mothers, 95 as male or fathers and 48 as couples; Blakeley‐Smith et al. (2021), and Ericson, Hesla, and Stadskleiv (2022) did not report these data. Three studies were conducted in the USA (Blakeley‐Smith et al. 2021; Singh et al. 2019, 2021), three in the Netherlands (Blankestein et al. 2019, 2020; Schuiringa et al. 2017), two in Canada (Heifetz and Dyson 2017; Picard, Morin, and De Mondehare 2014), one in Norway (Ericson, Hesla, and Stadskleiv 2022), one in Taiwan (Hu et al. 2010), one in England (Reid et al. 2016) and one in the Republic of Ireland and Northern Ireland (McMahon, Wilson, and Sharry 2023). The majority of adolescents were reported to have a borderline or mild intellectual disability. However, studies conducted in the Netherlands (i.e., Blankestein, 2019, 2020; Schuiringa et al. 2017) included adolescents with a Full Scale IQ of < 85 as these individuals can access intellectual disability services. Therefore, some participants did not have an intellectual disability based on this review's definition. These studies have been included in the review because of the small amount of relevant literature.

Study designs included four quantitative non‐randomised, controlled trials (Blankestein et al. 2019; Hu et al. 2010; Picard, Morin, and De Mondehare 2014; Singh et al. 2019) with one additional follow‐up study (Blankestein et al. 2020), three pre‐post designs (Blakeley‐Smith et al. 2021), two of which utilised a mixed methods approach (Ericson, Hesla, and Stadskleiv 2022; Heifetz and Dyson 2017), three quantitative randomised controlled trials (RCTs) (McMahon, Wilson, and Sharry 2023; Schuiringa et al. 2017; Singh et al. 2021) and one qualitative interview study (Reid et al. 2016). Five of the 12 studies included data on the experience of parents, through quantitative (Blakeley‐Smith et al. 2021; Picard, Morin, and De Mondehare 2014), qualitative (Heifetz and Dyson 2017; Reid et al. 2016) or quantitative and qualitative approaches (Ericson, Hesla, and Stadskleiv 2022). Six of the 10 interventions were reported as being manualised (Blakeley‐Smith et al. 2021; Blankestein et al. 2019, 2020; McMahon, Wilson, and Sharry 2023; Picard, Morin, and De Mondehare 2014; Schuiringa et al. 2017; Singh et al. 2019, 2020).

### 3.3 Quality of Reporting

As displayed in Tables 1, 3 and 6 an overall high level of intervention detail was provided, with scores ranging from 7 to 11 and eight of the 12 studies scoring 10 or higher on the TIDieR checklist. Tailoring, referring to how an intervention was planned to be personalised or adapted, was not reported for the vast majority (*n* = 9) of studies. Modifications, in terms of changes to the intervention that occurred during the study, were not reported for any studies. In the case of Multisystemic Therapy‐Intellectual Disabilities (MST‐ID) (Blankestein et al. 2019), modifications did not occur so as not to deviate from the treatment principles. Fidelity, referring to the extent to which an intervention was delivered in the way intended, or intervention adherence, was not reported for a minority (*n* = 2) of studies. The least comprehensively described intervention was reported by Reid et al. 2016; although the goals and structure of the intervention were discussed, details of exercises and discussions were lacking, which may be due to the intervention not being manualised.

As described in Table 9, the lowest‐scoring studies on the MMAT (Ericson, Hesla, and Stadskleiv 2022; Heifetz and Dyson 2017) used mixed‐methods approaches but lacked a clear rationale for this choice and did not effectively integrate study components. Other reasons for low scores on the MMAT included participants not representing the target population, unaccounted confounding variables, incomplete outcome data and inadequate qualitative data collection methods. These factors limit the conclusions that can be drawn about the effectiveness of these interventions. On average, parent well‐being interventions received higher ratings on the MMAT compared with parenting and interventions working through parents to influence adolescent outcomes.

### 3.4 Parenting Interventions

Two studies reported parenting interventions and both were RCTs (McMahon, Wilson, and Sharry 2023; Schuiringa et al. 2017), intervention characteristics are provided in Table 1, with details of effectiveness reported in Table 2. Parents Plus Special Needs (PPSN) and Standing Strong Together (SST) aimed to improve adolescent behaviour through improvements in parenting practises, covering similar topics such as positive communication and rules (McMahon, Wilson, and Sharry 2023; Schuiringa et al. 2017). In addition to parent training, PPSN incorporated mindfulness exercises and aimed to support families through parent‐only group discussion. Whereas, SST included cognitive behaviour therapy (CBT) and involved separate parent and adolescent sessions, adolescent sessions focused on recognising and managing emotions and parent sessions aimed to improve parent–adolescent interaction skills. Both interventions also taught parents self‐care practises.

Both interventions were manualised, utilised a group delivery format and were delivered by professionals with intervention training. In terms of the number of hours, SST was a more intensive intervention with 15 h each for parent and adolescent sessions, while PPSN involves 17.5 h of parent‐only sessions. Details of tailoring and modifications were unreported in both studies, and PPSN lacked an assessment of fidelity. PPSN was tailor‐made for parents of adolescents with intellectual disability, contrastingly, SST was adapted from existing interventions for children with disruptive behaviour disorders.

#### 3.4.1 Intervention Effectiveness

McMahon, Wilson, and Sharry (2023) assessed PSSN through a cluster RCT involving 24 disability services. In comparison to the waiting list group (*n* = 136), parents in the PPSN group (*n* = 141) reported improvements in parenting practices, parental self‐efficacy, problem behaviours and parental satisfaction post‐intervention. Improvements were maintained at the three‐month follow‐up. Although family adjustment had not improved post‐intervention for the PPSN group, significant improvements were found at the three‐month follow‐up. PPSN did not impact adolescent emotional problems. For SST, an RCT found effects in favour of the intervention group (*n* = 97) in comparison to care‐as‐usual (*n* = 72) for teacher‐reported adolescent externalising behaviour, parent‐reported positive parenting and the parent–child relationship (Schuiringa et al. 2017). However, adolescents in the intervention group reported more aggressive social cognitions post‐intervention.

#### 3.4.2 Summary

Both parenting interventions reported improvements in the intervention groups on measures of adolescent behaviour and parenting strategies. Despite both being RCTs, MMAT scores were low; reasons included incomplete outcome measures. For PSSN from pre‐ to post‐intervention 23.1% of data were missing (McMahon, Wilson, and Sharry 2023), and this was 17.16% for SST with a further 20.12% excluded from analyses due to an absence of socioeconomic and IQ data (Schuiringa et al. 2017). These parenting interventions did not measure parent experience.

**TABLE 1:** Characteristics of parenting interventions for parents of adolescents with intellectual disability using the TIDieR checklist (Hoffmann et al. 2014).

Study	Intervention name	Why	What (materials)	What (procedures)	Who provided	How	Where	When and how much	Tailoring	Modifications (during the study)	How well, planned (adherence or fidelity assessed?)	How well, actual (extent intervention delivered as planned)	TIDieR items reported
McMahon, Wilson, and Sharry (2023)	Parents Plus Special Needs (PPSN)	Group parenting interventions provide resources for parents to alleviate stress and enhance ability to cope, improve adolescent behaviour and to strengthen the family.	Manual‐based. Parent booklet.	Discussion with clinicians and other parents. Individual tasks, e.g., worksheets and mindfulness exercises.	Two trained facilitators from professionally qualified multi‐disciplinary team, e.g., psychologists, support workers and nurses.	Group, face‐to‐face.	23 disability services sites across the Republic of Ireland and Northern Ireland.	Seven weeks, two—two and a half hours. One three‐month follow‐up group session.	Not reported.	Not reported.	Facilitators required to complete session review forms and received two supervision sessions from a Parents Plus trainer and one group supervision session.	Not reported.	9/12
Schuiringa et al. (2017)	Standing Strong Together (SST)	Positive impact of behavioural training and CBT on externalising behaviour, adapted for families with adolescents with intellectual disability.	Manualised intervention based on the Utrecht Coping Power Program. (Van De Wiel, Matthys, Cohen‐Kettenis, & Van Engeland, 2003, 2007)	Adolescent sessions: recognising and managing feelings and problem‐solving. Parent sessions: parent–child interaction skills, practicing skills and self‐care.	Adolescents: group leader and a therapist. Parents: social worker and a group leader.	Group‐based, not specified if face‐to‐face. Between three to five adolescents.	Two Netherland treatment centres.	10, 90‐min parent sessions, alternate weeks. 12, 75‐min weekly child sessions.	Not reported.	Not reported.	One‐day training course provided by SST developers. Sessions audiotaped, 10% assessed.	Exercises: 75%, session parts: 85%, competence: 72%, enthusiasm: 68%.	10/12

Abbreviation: CBT, cognitive behavioural therapy.

**TABLE 2:** Effectiveness of parenting interventions for parents of adolescents with intellectual disability.

Study	Country	Study design	Participants	Intervention	Outcome measures	Analysis	Results	Total MMAT scores
McMahon, Wilson, and Sharry (2023)	Republic of Ireland and Northern Ireland	Cluster RCT	277 parents of adolescents with intellectual disability: PPSN: *n* = 141, waiting list: *n* = 136.	Parents Plus Special Needs	PAFAS (Sanders, Morawska, Haslam, Filus, and Fletcher, 2013), CAPES‐DD (Emser, Mazzucchelli, Christiansen, and Sanders, 2016), KPSS (James et al., 1985).	ANOVA to assess the impact of the intervention and t‐tests to determine the nature of significance.	Parent mental health/well‐being: Time and group interaction for parental satisfaction: *F*(1, 276) = 25.00, *p* < 0.001, $\eta_{p}^{2}$ = 0.08 with significant improvement in PPSN group: *t*(128) = −3.21, *p* < 0.003, *d* = 0.4. Parenting skills: Time and group interaction for parenting practices: *F*(1, 276) = 20.56, *p* < 0.001, $\eta_{p}^{2}$ = 0.07 and self‐efficacy: *F*(1, 235) = 11.70, *p* < 0.005, $\eta_{p}^{2}$ = 0.05, significant improvement in both in PPSN group *t*(141) = 4.88, *p* < 0.001, *d* = 0.4, *t*(128) = −12.78, *p* < 0.001, *d* = 0.3, respectively. No significant changes for family adjustment. Adolescent behaviour: Time and group interaction for problem behaviours: *F*(1, 275) = 5.60, *p* < 0.02, $\eta_{p}^{2}$ = 0.02, and prosocial behaviour: *F*(1, 265) = 6.30, *p* < 0.02, $\eta_{p}^{2}$ = 0.02. Significant improvement in problem behaviour in PPSN group: *t*(141) = 4.17, *p* < 0.001, *d* = 0.4. No significant changes in prosocial behaviour or emotional difficulties.	Quantitative RCT: 2/5
Schuiringa et al. (2017)	The Netherlands	RCT	169 parent/s and their adolescent with mild to borderline intellectual disability and externalising problems: Standing Strong Together intervention: *n* = 97, control: *n* = 72.	Standing Strong Together	CBCL/TRF (Verhulst, Van der Ende, and Koot, 1996). PDR (Chamberlain and Reid, 1987). SPT‐MID (Van Nieuwenhuijzen, Bijman, Lamberix, Wijnroks, and Matthys, 2001). NOBAGS (Huesmann and Guerra, 1997). APQ (Shelton, Frick, and Wootton, 1996) and some GPBS items (Van Leeuwen and Vermulst, 2004), PSI (De Brock, Vermulst, Gerris, and Abidin, 1992).	Repeated measures ANCOVAs to compare means from pre‐ to post‐intervention while controlling for demographic variables.	Adolescent behaviour: Externalising behaviour (TRF): F = 4.15, *p* = 0.045, *d* = 0.25. Aggressive social cognitions: F = 7.73, *p* = 0.01, *d* = −0.40. No significant intervention effect for externalising behaviour on the CBCL or PDR. Parenting skills: Positive parenting: F = 4.51, *p* = 0.04, *d* = 0.18. Negative Parenting: no significant intervention effect. Parent–adolescent relationship: F = 5.74, *p* = 0.02, *d* = 0.33.	Quantitative RCT: 3/5

Abbreviations: APQ, Alabama Parenting Questionnaire; CAPES‐DD, Child Adjustment and Parent Efficacy Scale‐Developmental Disability; CBCL/TRF, Child Behaviour Checklist/Teacher's Report Form; GPBS, Ghent Parental Behaviour Scale; KPSS, Kansas Parental Satisfaction Scale; NOBAGS, Normative Beliefs About Aggression Scale; PAFAS, Parenting and Family Adjustment Scale; PDR, Parent Daily Report; PSI, Parenting Stress Index (Dutch version); SPT‐MID, Social Problem Solving Test revised for children with Mild to Borderline Intellectual Disability.

### 3.5 Parent Well‐being

Five studies reported interventions that primarily focused on improving parent well‐being, these included one RCT (Singh et al. 2021), three non‐randomised controlled trials (Hu et al. 2010; Picard, Morin, and De Mondehare 2014; Singh et al. 2019) and one qualitative interview study (Reid et al. 2016). Four well‐being interventions incorporated mindfulness‐based approaches (Hu et al. 2010; Singh et al. 2019, 2021) and one intervention evaluated the use of psychoeducation (Picard, Morin, and De Mondehare 2014). Evaluation of parent well‐being interventions are provided in Table 3; details of effectiveness are reported in Table 4 and information concerning parent experience is displayed in Table 5.

Two studies evaluated Mindfulness‐Based Positive Behaviour Support (MBPBS), an intervention previously developed for caregivers of individuals with a variety of disorders and conditions displaying challenging behaviour (Singh et al. 2016). Based on a protocol by Singh et al. (2020), MBPBS combines mindfulness and positive behaviour support (PBS) to target parents' psychological needs and manage adolescent challenging behaviour (Singh et al. 2019, 2021).

Three interventions were specifically developed for parents of adolescents with intellectual disability (Hu et al. 2010; Picard, Morin, and De Mondehare 2014; Reid et al. 2016). Two of these studies implemented short parent well‐being workshops and covered topics of stress and thought processes/positive thinking; Reid et al. (2016) incorporated mindfulness and acceptance and commitment therapy concepts with the aim to improve parent emotional well‐being. In comparison, Hu et al. (2010) provided stress management and relaxation strategies through a parent workshop and stress‐relief booklet to reduce parent stress. The third intervention also aimed to improve parental stress but implemented a psychoeducational programme to provide parents with information on topics such as support services, parenting skills, relationships and legal proceedings (Picard, Morin, and De Mondehare 2014).

All parent well‐being interventions were delivered in a face‐to‐face group format, although Reid et al. (2016) did not report if groups were face‐to‐face. The two well‐being workshops were both provided by clinical psychologists or psychiatrists, and MBPBS and the psychoeducational programme were delivered by experienced individuals. MBPBS was the most intensive intervention, in terms of the number of hours, incorporating three training days and 30 weeks of parents implementing learned strategies. The least intensive interventions were the two well‐being workshops, totalling 2 h (Hu et al. 2010) and 8 h (Reid et al. 2016), these interventions also received the lowest TIDieR scores, both not reporting measures of fidelity. None of these well‐being interventions included details of tailoring or modifications.

#### 3.5.1 Intervention Effectiveness

Through a RCT, MBPBS (*n* = 65) was found to be more effective than mindfulness‐based practices (*n* = 65) or PBS alone (*n* = 65) in improving parent psychological stress, adolescent aggression, adolescent disruptive behaviour and adolescent compliance (‘responding to his mother's requests in a socially appropriate manner within an acceptable timeframe’ (Singh et al. 2014, 648)), with improvements maintained 3 years post‐intervention (Singh et al. 2021). Singh et al. (2019) compared the effects of MBPBS for parents of adolescents with an intellectual disability (*n* = 55) and parents of autistic adolescents without an intellectual disability (*n* = 55) through a non‐randomised control study. Considering the results from the intellectual disability group, adolescent aggression, disruptive behaviour and compliant behaviour improved and parent stress significantly decreased post‐intervention. However, both MBPBS studies only included mothers and the exclusion of fathers limits the generalisability of the findings.

In terms of the parent well‐being workshops, Hu et al. (2010) conducted a non‐randomised control trial and found that parent depressive stress levels significantly improved for participants who received the workshop and stress‐relief booklet (*n* = 31) in comparison to a control group (*n* = 46). Depressive stress, defined as the existence of physical and emotional symptoms associated with depression or stressful circumstances, was measured through the Taiwanese Depression Questionnaire (Hu et al. 2010; Lee et al. 2000). A qualitative interview study conducted by Reid et al. (2016) identified themes of ‘new ways of seeing and being’ and ‘positive changes’ reported by parents following participation in the well‐being workshops. Another theme of ‘looking to the future’ was identified as parents reported the development of life‐changing techniques and the beginning of a new mindfulness journey. Parents also reported improvements in stress, self‐care and parent–adolescent interactions.

A non‐randomised control trial conducted by Picard, Morin, and De Mondehare (2014) compared a psychoeducational programme (*n* = 25) to a self‐help group (*n* = 5) and found no significant differences in parental stress, depression or anxiety. Parents participating in the psychoeducational programme reported increased adolescent distractibility/hyperactivity, while parents in the self‐help group reported decreases.

#### 3.5.2 Parent Experience

Regarding parent experience of participating in the intervention, bespoke quantitative evaluation forms showed that the psychoeducation group was favoured across most variables including helpfulness, motivation to get support and tips for parent–child interactions (Picard, Morin, and De Mondehare 2014). Further, all psychoeducation sessions were reported as more than moderately helpful. Through semi‐structured interviews, Reid et al. (2016) identified the theme of ‘a unique group’, highlighting the difference between this well‐being workshop, previous experiences and preconceptions of mindfulness. Parents also emphasised the importance of meeting other parents.

#### 3.5.3 Summary

All studies that incorporated mindfulness or relaxation strategies reported improvements in parents' mental health or well‐being. However, the psychoeducation intervention did not find any significant differences between groups in parent mental health outcomes (Picard, Morin, and De Mondehare 2014). Both studies evaluating MBPBS reported sustained improvements in adolescent behaviour over time. However, no improvements in adolescent behaviour were reported in the psychoeducation intervention, with some evidence of adolescent behaviour worsening. All three non‐randomised studies (Hu et al. 2010; Picard, Morin, and De Mondehare 2014; Singh et al. 2019) received scores of two out of five on the MMAT; reasons included studies not accounting for confounding variables and unrepresentative samples. These three studies were also limited based on the small sample size and the lack of randomization. The RCT (Singh et al. 2021) and qualitative interview study (Reid et al. 2016) received the highest MMAT scores across all studies, suggesting these were well conducted. Studies that measured parent experience reported positive responses in terms of helpfulness (Picard, Morin, and De Mondehare 2014) and providing unique and valuable support (Reid et al. 2016).

**TABLE 3:** Characteristics of parent well‐being interventions for parents of adolescents with intellectual disability using the TIDieR checklist (Hoffmann et al. 2014).

Study	Intervention name	Why	What (materials)	What (procedures)	Who provided	How	Where	When and how much	Tailoring	Modifications (during the study)	How well, planned (adherence or fidelity assessed?)	How well, actual (extent intervention delivered as planned)	TIDieR items reported
Hu et al. (2010)	Stress‐relief program	Stress management strategies and relaxation techniques for parents of adolescents with intellectual disability.	Stress‐relief booklet.	Identify the symptoms and cause of stress, management, relaxation and social support.	Senior psychiatrist.	Group, face‐to‐face.	Taipei, Taichung and Yuling.	Two‐hour workshop and a stress relief booklet.	Not reported.	Not reported.	Not reported.	Not reported.	8/12
Picard, Morin, and De Mondehare (2014)	Psychoeducational programme	Increase parent support and access to services to positively impact the well‐being of parents of adolescents with intellectual disability.	150‐page manual. Reference document and readings.	Parents were provided with information and advice. Self‐help comparison group: discussion on the same topics minus introduction to intellectual disability.	Principal investigator had an understanding of intellectual disability and services.	Group, face‐to‐face, four to eight participants and five in self‐help group.	Quebec.	10, two‐hour weekly sessions. Self‐help: five, two‐hour sessions, two‐week intervals.	Not reported.	Not reported.	20% of sessions recorded. Principal investigator supervised.	All topics covered.	10/12
Reid et al. (2016)	Parent Well‐Being Workshops	Target the well‐being of parents to support adolescents with intellectual disability and behavioural problems.	Not reported.	Key concepts of Acceptance and Commitment Therapy and metaphors (Smith and Gore, 2012). Exercises and discussion to incorporate skills.	Two clinical psychologists.	Group format, does not specify if face‐to‐face.	Ealing Intensive Therapeutic and Short Breaks Service.	Two, four‐hour workshops held one week apart.	Not reported.	Not reported.	Not reported.	Not reported.	7/12
Singh et al. (2019)	Mindfulness‐Based Positive Behaviour Support (MBPBS)	Mindfulness reduces parent stress. Positive Behaviour Support aids the management of challenging adolescent behaviour.	Adapted MBPBS protocol (Singh et al. 2020). Positive Behaviour Support plans.	Mindfulness training, developing positive behaviour intervention and support plans and daily meditation.	Experienced in meditation, behaviour analysis and interventions.	Group format, face‐to‐face.	In the community.	Three‐day training sessions and 30 weeks of implementation.	Not reported but mothers developed positive behaviour intervention and support plans.	Not reported.	A random 10–12 min per hour of training videotaped and rated. Attendance recorded. Daily log of meditation.	Fidelity of training components: 100%. Attendance: 100%. Meditation: 18.23 min.	10/12
Singh et al. (2021)	Mindfulness‐Based Positive Behaviour Support (MBPBS)	Mindfulness relieves parent stress, combining with Positive Behaviour Support aids the management of adolescent behaviour.	Streamlined protocol (Singh et al. 2019). Materials to create Positive Behaviour Support plans.	MBPBS: mindfulness (meditation), behaviour plans and skills. Mindfulness‐based: mindfulness only. Positive Behaviour Support: positive behaviour support training.	Experienced in meditation, behavioural analysis and interventions.	Face‐to‐face or via video or phone. Five to 13 per group.	Locations close to participants or via telemedicine.	Three‐day training, 30 weeks of implementation. 20 min a day meditation suggested.	Not reported but mothers developed positive behaviour intervention and support plans.	Not reported.	Sessions videotaped for 15 min each hour per day at random. Attendance monitored. Daily log of meditation.	Structural (what) and process (how) fidelity: 100%. Attendance: 100%. Meditation: 20–24 min.	10/12

Abbreviation: Mindfulness‐based Positive Behaviour Support (MBPBS).

**TABLE 4:** Effectiveness of parent well‐being interventions for parents of adolescents with intellectual disability.

Study	Country	Study design	Participants	Intervention	Outcome measures	Analysis	Results	Total MMAT scores
Hu et al. (2010)	Taiwan	Non‐randomised controlled trial	77 primary caregivers of adolescents with intellectual disability. Intervention: *n* = 31, control: *n* = 46.	Stress‐relief program	TDQ (Lee et al. 2000).	Descriptive and paired t‐test to compare means between groups.	Parent mental health/well‐being: Lack of depressive stress increased in the intervention (41.9% to 64.5%), control decreased (50% to 47.8%). Depressive stress levels: *t* = −2.144, *p* = 0.040.	Quantitative non‐randomised: 2/5
Picard, Morin, and De Mondehare (2014)	Canada	Non‐randomised controlled trial	30 parents of an adolescent with intellectual disability, intervention: *n* = 25, self‐help: *n* = 5.	Psychoeducational programme	BDI (Beck, Steer, and Brown, 2005), BAI (Freeston, Ladouceur, Thibodeau, Gagnon, and Rhéaume, 1994), PSI (Bigras, LaFreniere, and Abidin, 1996).	ANOVA‐type and descriptive to compare means between groups.	Parent mental health//well‐being: No significant differences. Adolescent behaviour: Distractibility/hyperactivity subscale: (F(1, 27) = 4.285, *p* = 0.048): increased in the intervention, decreased in the comparison. No other significant differences in child domains of PSI.	Quantitative non‐randomised: 2/5
Reid et al. (2016)	England	Qualitative Interview	Seven parents of autistic adolescents with severe intellectual disability and challenging behaviour.	Parent well‐being workshops	Semi‐structured interviews.	Thematic analysis (Braun and Clarke, 2006) to identify themes.	Parent mental health/well‐being: ‘value‐led changes’: less stress and more self‐care. Parenting skills: ‘New ways of seeing and being’: changed perceptions and use of mindfulness. ‘Positive changes’: alternative responses to approaching difficulties. ‘Looking to the future’: personal practice. Parent–adolescent relationship: improved interactions.	Qualitative: 5/5
Singh et al. (2019)	USA	Non‐randomised controlled trial	55 mothers of adolescents with mild intellectual disability (and 55 mothers of autistic adolescents‐results not considered).	Mindfulness‐Based Positive Behaviour Support	PSS‐10 (Cohen, Kamarck, and Mermelstein, 1983) and app recording events.	Mixed‐model ANOVA to compare effects of group, time and interaction. Phi coefficient to assess the control and intervention overlap.	Parent mental health/well‐being: Significant effect of assessment time: F(2,89) = 637.82, *p* < 0.001 (η2 = 0.935). Adolescent behaviour: Aggression: phi = 0.66, *p* < 0.001, disruption: phi = 0.87, *p* < 0.001, compliance: phi = 0.87, *p* < 0.001.	Quantitative non‐randomised: 2/5
Singh et al. (2021)	USA	RCT	195 mothers of autistic adolescents with intellectual disability, MBPBS: *n* = 65, MB: *n* = 65, PBS: *n* = 65.	Mindfulness‐Based Positive Behaviour Support	PSS‐10 (Cohen et al. 1983) and app recording events.	Two‐level ANCOVA to compare effects of group, time and interaction. Multiple linear regression to assess behaviour outcomes overtime.	Parent mental health/well‐being: Effect of group *F*(2,170) = 76.64, *p* < 0.001, η2 = 0.47, time and group interaction *F*(10,170) = 196.73, *p* < 0.001, η2 = 0.69. Adolescent behaviour: Aggression: time effect = 28% of reduction (R^2^ = 0.28; β = − 0.53; *p* < 0.001), MBPBS = additional 5% (R^2^ = 0.05; β = − 0.23; *p* = 0.002). Disruptive: time effect in all groups, 35% of reduction (R^2^ = 0.35; β = − 0.59; *p* < 0.001), MBPBS = additional 4% (R^2^ = 0.04; β = − 0.20; *p* = 0.004). Compliance: time effect = 27% of the increase (R^2^ = 0.27; β = 0.52; *p* < 0.001), additional 8% = MBPBS (R^2^ = 0.08; β = 0.28; *p* < 0.001). Meditation effect after controlling for time and intervention type (R^2^ = 0.27; β = 0.85; *p* < 0.001).	Quantitative RCT: 4/5

Abbreviations: BAI, Beck Anxiety Inventory; BDI, Beck Depression Inventory; PSI, Parenting Stress Index; PSS‐10, Perceived Stress Scale‐10; TDQ, Taiwanese Depression Questionnaire.

**TABLE 5:** Parent experience of parent well‐being interventions for parents of adolescents with intellectual disability.

Study	No. of caregivers	Intervention	Data collection	Analysis	Quantitative outcomes	Qualitative outcomes
Picard, Morin, and De Mondehare (2014)	30	Psychoeducational programme	Evaluation sheet after each session: Likert scales: ‘not at all helpful’ (1) – ‘very helpful’ (5), ‘not at all’ (1)—'very much’ (5) and ‘much reduced’ (1)—‘much increased’ (5).	Descriptive analyses.	On average, all topics covered in the 10 sessions were scored above moderately helpful. Parents rated the session on financial and legal aspects as the most helpful and the school session the least helpful. Both the intervention and comparison group were positively evaluated. Comparisons between the two groups suggest that mean differences favoured the intervention group for 16 of the 25 variables.	
Reid et al. (2016)	5	Parent well‐being workshops	Semi‐structured interview with parents.	Thematic analysis (Braun and Clarke, 2006).		Parents described the intervention as unique and value was placed on meeting people with similar experiences and developing cohesion and hope. Parents reported that the intervention was different to previous experiences of support, enabled parents to discuss aspects previously undisclosed and challenged expectations.

### 3.6 Interventions Working Through Parents to Influence Adolescent Outcomes

Five studies reported interventions working through parents to influence adolescent outcomes and consisted of one non‐randomised controlled trial (Blankestein et al. 2019) with one additional follow‐up (Blankestein et al. 2020) and three pre‐post designs (Blakeley‐Smith et al. 2021; Ericson, Hesla, and Stadskleiv 2022; Heifetz and Dyson 2017). All interventions included an adolescent component but adopted a variety of approaches to target adolescent behavioural and emotional problems including targeting multiple systems surrounding the adolescent (Blankestein et al. 2019), CBT (Blakeley‐Smith et al. 2021), psychoeducation (Ericson, Hesla, and Stadskleiv 2022) and mindfulness (Heifetz and Dyson 2017). Descriptions of interventions working through parents to influence adolescent outcomes interventions are provided in Table 6, with details of effectiveness reported in Table 7 and parent experience data displayed in Table 8.

Three studies were adaptations of manualised family‐based interventions (Blakeley‐Smith et al. 2021; Blankestein et al. 2019, 2020). Two of these studies looked at the adaptation of MST for adolescents with intellectual disability. Blakeley‐Smith et al. (2021) adapted a CBT group programme, Facing Your Fears (Reaven et al. 2011). Both interventions made adaptations to support adolescents' cognitive and communication abilities including language use and visual cues. However, MST targeted adolescent antisocial behaviour with parent empowerment and parental skills as focal points (Henggeler and Schaeffer 2016), whereas, Facing Your Fears aimed to improve the regulation and management of anxiety in autistic adolescents with intellectual disability; parent sessions included psychoeducation on anxiety and adolescents' problem behaviour.

Two studies involved interventions developed specifically for parents of adolescents with intellectual disability (Ericson, Hesla, and Stadskleiv 2022; Heifetz and Dyson 2017). Both interventions aimed to improve social behaviour/inclusion and adolescent coping or emotional regulation. Ericson, Hesla, and Stadskleiv (2022) implemented a psychoeducation programme, the Super Control Project, with parallel groups for adolescents and parents, covering topics such as intellectual disability diagnosis, social inclusion, support, parenting and transition to adulthood. Contrastingly, Calming Thoughts and Calming Minds incorporated a mindfulness approach; parent sessions aimed to help parents understand, use and support the mindfulness skills being taught to their adolescents (Heifetz and Dyson 2017).

MST‐ID was the only intervention that utilised an individual parent–adolescent format, all other interventions implemented a group format with both parents and adolescents (Blankestein et al. 2019, 2020). Although Blankestein et al. (2019, 2020) did not describe session frequency or duration, MST typically involves 60–100 h of contact, with MST adaptations often involving more (Henggeler and Schaeffer 2016). Therefore, MST‐ID can be described as the most intensive intervention. Facilitators varied from individuals with experience in the area of intllectual disability (Blankestein et al. 2019, 2020; Heifetz and Dyson 2017) to clinical psychologists (Ericson, Hesla, and Stadskleiv 2022), Blakeley‐Smith et al. (2021) did not report details of facilitator background. All interventions were well described, scoring 10 or above on the TiDieR checklist.

#### 3.6.1 Intervention Effectiveness

The non‐randomised controlled trial conducted by Blankestein et al. (2019) found that parents in the MST‐ID group (*n* = 55) reported a reduction in parent‐reported adolescent rule‐breaking behaviour at the 6‐month follow‐up, which was maintained at 18 months (Blankestein et al. 2020). In comparison to the MST group (*n* = 73), MST‐ID resulted in greater improvements in parenting skills and changes in adolescent problem behaviours. No significant differences were found for parent stress, adolescent externalising problems or rule‐breaking behaviour outcomes.

The three remaining interventions were pre‐post designs. In the adapted Facing Your Fears intervention with 23 adolescents and their parents, parents reported a reduction in adolescent anxiety and lethargy but no other significant changes in adolescent behaviour post‐intervention (Blakeley‐Smith et al. 2021). In the Super Control Project, involving 24 adolescents and their parents, parents and adolescents reported improvements in adolescent practical skills and mental health. Teachers and adolescents also reported improvements in social outcomes (Ericson, Hesla, and Stadskleiv 2022). However, a less stringent level of statistical significance was used (0.10), increasing the likelihood of a type one error. The Calming Thoughts and Calming Minds Program was evaluated with eight adolescents and their 10 parents; adolescents on average reported feeling happier, more relaxed and less worried post‐intervention and parents reported higher levels of adolescent social behaviour and an increase in mindful parenting (Heifetz and Dyson 2017).

#### 3.6.2 Parent Experience

Three interventions working through parents to influence adolescent outcomes also measured parent experience. Facing Your Fears (Blakeley‐Smith et al. 2021) and the Super Control Project (Ericson, Hesla, and Stadskleiv 2022) were positively evaluated for their content, format and delivery. Parents reported that the Super Control Project (Ericson, Hesla, and Stadskleiv 2022) and Calming Thoughts and Calming Minds (Heifetz and Dyson 2017) interventions had a positive impact on their understanding. However, the short qualitative data collection methods used for evaluating parent experience of the Super Control Project (semi‐structured forms) and Calming Thoughts and Calming Minds (qualitative survey), may not be sufficient to obtain in‐depth insight, further, analysis of these data was unclear (Ericson, Hesla, and Stadskleiv 2022; Heifetz and Dyson 2017). In addition to Likert ratings, parents were invited to provide qualitative feedback on the Facing Your Fears programme (Blakeley‐Smith et al. 2021), however, these data were not reported; integrating qualitative data would be beneficial to generate a deeper insight.

#### 3.6.3 Summary

Despite a diverse range of approaches, all interventions working through parents to influence adolescent outcomes reported improvements in adolescent behaviour and/or emotion. Interventions that involved the development of parenting skills reported improvements in parenting practices (Blankestein et al. 2019; Heifetz and Dyson 2017). None of the interventions met the full MMAT quality assessment criteria, reasons for this included missing outcome data, for example, in MST‐ID, 58% of families were missing a least one follow‐up interview (Blankestein et al. 2020) and Ericson, Hesla, and Stadskleiv (2022) reported 13% missing parent outcome data with a further 13% excluded. Participants also frequently did not represent the target population or confounding variables were not accounted for. The majority of these studies adopted a pre‐post design and are therefore limited by the lack of a control group. All studies that included a measure of parent experience of the intervention reported positive responses, particularly in terms of format and perceived impact.

**TABLE 6:** Characteristics of interventions working through parents to influence the outcomes of adolescents with intellectual disability using the TIDieR checklist (Hoffmann et al. 2014).

Study	Intervention name	Why	What (materials)	What (procedures)	Who provided	How	Where	When and how much	Tailoring	Modifications (during the study)	How well, planned (adherence or fidelity assessed?)	How well, actual (extent intervention delivered as planned)	TIDieR items reported
Blakeley‐Smith et al. (2021)	Facing Your Fears adaptation	CBT is effective at improving emotional regulation and anxiety. Adapted a family‐focused CBT intervention for autistic adolescents with intellectual disability and anxiety.	Facing Your Fears Manual (Reaven et al. 2011). Schedules and checklists.	CBT concepts, identify anxiety and problem behaviour, and aid the transition from parent‐supported to adolescent‐implemented.	Two facilitators but no further details.	Group format, two to four adolescents and their parents. Unclear if face‐to‐face.	Not reported.	14 sessions, 45–60 min, three parent‐only sessions, 11 parent–adolescent sessions.	Tailored exposures. Adaptations for language abilities.	Not reported.	Attendance recorded.	82.6% completed the intervention and attended 94% of sessions.	10/12
Blankestein et al. (2019, 2020)	Multisystemic Therapy‐Intellectual Disabilities (MST‐ID)	Multisystemic Therapy (MST) can reduce externalising behaviour. Adapted for families with an adolescent with intellectual disability.	MST manual (Henggeler, Schoenwald, Borduin, Rowland, and Cunningham, 2009).	Strengthens family support systems and parental skills. Develops a supportive environment to encourage adaptive behaviour.	Specialised in intellectual disability and higher education in social sciences.	In person—individual (parent and adolescent).	Family home.	MST‐ID mean duration = 5.1 months (range: 2 to 8 months).	Not reported but tailoring based on the referred problem behaviour.	Not reported.	Five‐day training, supervision, consultations, and booster sessions. Therapist adherence to principles scored.	TAM‐R MST‐ID mean = 4.35 (Standard Deviation = 0.56) Scale: 1–5 (5 very much).	10/12
Ericson, Hesla, and Stadskleiv (2022)	Super Control Project	More knowledge for parents and adolescents may improve adolescents' behavioural and emotional outcomes. Intervention focus on understanding of diagnosis, social network and overcoming challenges.	Olsson, Bergqvist, and Myrberg (2007): materials and videos https://nrksuper.no. Quizzes.	Adolescent sessions: life‐situations, diagnosis and well‐being. Parent sessions: family life and parenting. Websites and readings provided. Parent topics from Picard, Morin, and De Mondehare (2014).	Adolescents: two clinical psychologists. Parents: clinical psychologist and a social worker.	Group format, face‐to‐face, three to eight adolescents.	Norway's paediatric habilitation centres. Exact location unclear.	Parallel adolescent and parent groups. Six, two‐hour weekly sessions.	Materials adapted based on cognitive capacities. Parent topics tailored to group.	Not reported.	Attendance was monitored.	Completed by 22/23 of the families.	11/12
Heifetz and Dyson (2017)	Calming Thoughts and Calming Minds	Teaching mindfulness‐based practices to adolescents and their parents to improve adolescent emotional regulation.	Feelings cards, emotional thermometer, videos and recordings.	Adolescent sessions: defining mindfulness, how and when to practice. Parent sessions: generalising mindfulness skills and using mindfulness‐based skills themselves.	Two doctoral students with experience with intellectual and development disabilities and mindfulness.	Group format, unclear if face‐to‐face.	Surrey Place Centre, Toronto, Canada.	Eight sessions, 90 min each over 3 months: two joint parent–adolescents, five adolescent only, one in parallel.	Not reported.	Not reported.	Supervision after each session and session outlines followed closely. Attendance monitored.	6/8 adolescents and 8/10 parents completed the programme.	10/12

Abbreviations: CBT, Cognitive Behavioural Therapy; TAM‐R, Therapist Adherence Measure‐Revised (Henggeler, Borduin, Schoenwald, Huey, and Chapman, 2016).

**TABLE 7:** Effectiveness of interventions working through parents to influence the outcomes of adolescents with intellectual disability.

Study	Country	Study design	Participants	Intervention	Outcome measures	Analysis	Results	Total MMAT scores
Blakeley‐Smith et al. (2021)	USA	Pre‐post	23 autistic adolescents, also diagnosed with intellectual disability and anxiety and their parents.	Facing Your Fears adaptation	ADAMS (Esbensen, Rojahn, Aman, and Ruedrich, 2003), SCARED‐P (Birmaher et al. 1999), FSSC‐R (Ollendick, 1983), ABC (Aman, 2012).	Descriptive statistics and linear mixed models to compare baseline and follow‐up scores.	Adolescent behaviour and emotion: ADAMS total: *F*(1,22.55) = 20.89, *p* < 0.0001, ωp^2^ = 0.45, all ADAMS subscales = significant change. SCARED‐P total: *F*(1,21.09) = 4.92, *p* = 0.038, ωp^2^ = 0.14. Separation anxiety subscale: ωp^2^ = 0.18, *p* = 0.023. No significant changes on other SCARED‐P subscales. FSSC‐R: *F*(1,17.60) = 6.01, *p* = 0.025, ωp^2^ = 0.20. ABC: Lethargy subscale, *F*(1,18.20) = 4.22, *p* = 0.05, ωp^2^ = 0.14. No significant changes on other ABC subscales: irritability, stereotypy, hyperactivity and inappropriate speech.	Quantitative non‐randomised: 3/5
Blankestein et al. (2019)	The Netherlands	Non‐randomised controlled study	128 primary caregivers and their adolescent with antisocial or delinquent behaviour and intellectual disability: MST‐ID (*n* = 55), standard MST (*n* = 73).	MST‐ID vs. MST	CBCL and YSR (Achenbach and Rescorla, 2001), OBVL (Vermulst, Kroes, De Meyer, Nguyen, and Veerman, 2012).	ANOVAs, regression analysis and logistic regression to estimate treatment effects. Propensity score used to adjust for allocation bias.	Adolescent behaviour: MST‐ID rule‐breaking F(1, 33) = 13.59, *p* < 0.01. MST‐ID vs. MST 6‐month follow‐up: externalising behaviour *B* = ‐3.991, (non‐significant difference), rule breaking *B* ‐0.496, (non‐significant difference), changes in problem behaviour: 93.3% vs. 78.8% (therapist reported), RR = 1.149 (significant change). Parent well‐being: MST‐ID vs. MST 6‐month follow‐up: parenting stress: *B* = ‐0.274, (non‐significant difference). Parenting skills: Therapist reported: MST‐ID: 93.3% vs. MST = 75.8%, RR = 1.232 (significant change).	Quantitative non‐randomised: 3/5
Blankestein et al. (2020)	The Netherlands	Pre‐post, 18 month follow‐up.	55 primary caregivers and their adolescent with antisocial or delinquent behaviour and intellectual disability.	MST‐ID	CBCL (Achenbach and Rescorla, 2001).	T‐tests and chi‐square to assess change over time. Missing data imputed using PMM.	Adolescent behaviour: rule‐breaking: Start‐end: t = −3.296, *p* = 0.001, d = −0.44, start‐18 months: t = −2.119 *p* = 0.034, d = −0.29.	Quantitative non‐randomised: 2/5
Ericson, Hesla, and Stadskleiv (2022)	Norway	Pre‐post	24 adolescents with mild intellectual disability and their parent/s.	Super Control Project	Questionnaire, Likert scale, 1 (disagree) to 5 (agree). Talking Mats: 1 (disagree) to (3 = always agree).	Mean scores, Cohen's d and paired sample t‐test to assess the change in mean score from pre‐ to post‐intervention.	Adolescent behaviour and emotion: Parent reports: practical skills, *t*(16) = 2.60, *p* < 0.05), *d* = 0.34 and mental health, *t*(16) = 1.77, *p* < 0.10, *d* = 0.36. Teacher reports: social adjustment/inclusion, *t*(10) = 3.19, *p* < 0.01, *d* = 0.38. Adolescent reports: practical skills, *t*(7) = 3.31, *p* = < 0.05, *d* = 0.93, social functioning, *t*(7) = 3.37, *p* < 0.05, *d* = 0.83 and mental health, *t*(7) = 2.21, *p* < 0.10, *d* = 0.62.	Qualitative: 0/5 Quantitative non‐randomised: 4/5 Mixed‐methods: 0/5
Heifetz and Dyson (2017)	Canada	Pre‐post	10 parent/s and their eight adolescents with mild intellectual disability.	Calming Thoughts and Calming Minds	Weekly Emotional Check‐ins: 5‐point Likert Scale‐higher score = more positive, e.g., 1 (very sad), 5 (very happy). IEM‐P (Duncan, 2007), CSBQ (Luteijn, Jackson, Volkmar, and Minderaa, 1998).	Descriptive data to see changes pre‐ to post‐intervention.	Adolescent behaviour and emotion: Happier M = 3.98 (0.63) vs. M = 4.30 (0.83), more relaxed M = 3.42 (1.05) vs. M = 3.84 (1.30), less worried M = 3.98 (0.63) vs. M = 4.30 (0.83) and more social behaviours M = 1.91 (0.85) vs. M = 2.14 (0.61). Parenting skills: More mindful parenting strategies: M = 10.25 (0.57) vs. M = 11.75 (1.69) and more mindful parenting: Awareness and attention: M = 3.39 (0.43) vs. M = 3.95 (0.48); Non‐judgement: M = 3.71 (0.36) vs. M = 4.27 (0.49); and Non‐reactivity: M = 3.14 (0.47) vs. M = 3.53 (0.90).	Qualitative: 1/5 Quantitative non‐randomised: 1/5 Mixed‐methods: 1/5

Abbreviations: ABC, Aberrant Behaviour Checklist; ADAMS, Anxiety, Depression, and Mood Scale; CBCL, Child Behaviour Checklist; CSBQ, Children's Social Behaviour Questionnaire; FSSC‐R, Fear Survey Schedule for Children‐Revised; IEM‐P, Interpersonal Mindfulness in Parenting; MST, Multisystemic Therapy; MST‐ID, Multisystemic Therapy‐Intellectual disability; OBVL, Opvoedingsbelasting Vragenlijst (Dutch Parenting Burdening Questionnaire); PMM, Predictive Mean Matching method; SCARED‐P, Screen for Child Anxiety and Related Emotional Disorders – Parent; YSR, Youth Self Report.

**TABLE 8:** Parent experience of interventions working through parents to influence the outcomes of adolescents with intellectual disability.

Study	No. of caregivers	Intervention	Data collection	Analysis	Quantitative outcomes	Qualitative outcomes
Blakeley‐Smith et al. (2021),	23	Facing Your Fears Adaptation	Questionnaire: Likert scale 0–5 (5 = extremely satisfied).	Descriptive analysis.	Mean acceptability ratings = 4.56. Mean of 4 or higher on 94.7% of ratings across six domains: somatic management, emotion regulation, cognitive strategies, exposure, creation of daily routines and group format.	
Ericson, Hesla, and Stadskleiv (2022)	21	Super Control Project	Semi‐structured evaluation forms. Likert scale 0–5 (5 = very relevant).	Descriptive analysis, qualitative analysis unclear.	Of the eight topics covered, parents rated all of these as relevant. The most highly rated topics included mental health, social welfare and education. The topic rated as less relevant was driver's licence.	12 parents reported the group created a generous climate to share and learn from one another. Social support and topic discussion were positively described. Parents reported obtaining information on target topics. Group format, climate, and quality of speakers were positively rated.
Heifetz and Dyson (2017)	8	Calming Thoughts and Calming Minds	Parent qualitative survey.	Qualitative analysis unclear.		Two key themes: (1) ‘Insight both parents and youths gained’: ‘…to step back and watch my child learn and use this technique. It brought to my attention some of his anxieties and gave me a better appreciation of his struggles. I found it helpful for me as well to use the techniques and feed off each other.’ (p. 451) 2) ‘More groups and more sessions’: ‘This is a fantastic group and I think there needs to be more of this’. (p. 451)

**TABLE 9:** Quality assessment of included studies using the MMAT (Hong et al. 2018).

	Screening questions		Method‐specific quality‐assessment questions					
Study	S1. Are there clear research questions?	S2. Do the collected data allow to address the research questions?	1. Qualitative					MMAT total score
			1.1 Is the qualitative approach appropriate to answer the research question?	1.2 Are the qualitative data collection methods adequate to address the research question?	1.3 Are the findings adequately derived from the data?	1.4 Is the interpretation of results sufficiently substantiated by the data?	1.5 Is there coherence between qualitative data sources, collection, analysis and interpretation?	
Ericson, Hesla, and Stadskleiv (2022)^a^	Yes	Yes	Can't tell	No	Can't tell	Yes	No	1/5
Heifetz and Dyson (2017)^a^	Yes	Yes	Can't tell	No	Can't tell	Yes	No	1/5
Reid et al. (2016)	Yes	Yes	Yes	Yes	Yes	Yes	Yes	5/5

			2. Quantitative randomised‐controlled trials					
			2.1. Is randomization appropriately performed?	2.2. Are the groups comparable at baseline?	2.3. Are there complete outcome data?	2.4. Are outcome assessors blinded to the intervention provided?	2.5 Did the participants adhere to the assigned intervention?	
McMahon, Wilson, and Sharry (2023)	Yes	Yes	Yes	Yes	No	No	Can't tell	2/5
Schuiringa et al. (2017)	Yes	Yes	Yes	Yes	No	No	Yes	3/5
Singh et al. (2021)	Yes	Yes	Yes	Yes	Yes	No	Yes	4/5

			3. Quantitative non‐randomised					
			3.1 Are the participants representative of the target population?	3.2 Are measurements appropriate regarding both the outcome and intervention (or exposure)	3.3 Are there complete outcome data?	3.4. Are the confounders accounted for in the design and analysis?	3.5. During the study period, is the intervention administered (or exposure occurred) as intended?	
Blakeley‐Smith et al. (2021)	Yes	Yes	Yes	Yes	Yes	No	Can't tell	3/5
Blankestein et al. (2019)	Yes	Yes	No	Yes	No	Yes	Yes	3/5
Blankestein et al. (2020)	Yes	Yes	No	Yes	No	No	Yes	2/5
Ericson, Hesla, and Stadskleiv (2022)^a^	Yes	Yes	Yes	No	No	No	Can't tell	1/5
Heifetz and Dyson (2017)^a^	Yes	Yes	Can't tell	Yes	No	No	No	1/5
Hu et al. (2010)	Yes	Yes	Can't tell	Yes	Yes	No	Can't tell	2/5
Picard, Morin, and De Mondehare (2014)	Yes	Yes	No	Yes	Can't tell	No	Yes	2/5
Singh et al. (2019)	Yes	Yes	No	Yes	No	No	Yes	2/5

			5. Mixed‐methods					
			5.1. Is there an adequate rationale for using a mixed methods design to address the research question?	5.2. Are the different components of the study effectively integrated to answer the research question?	5.3. Are the outputs of the integration of qualitative and quantitative components adequately interpreted?	5.4. Are divergences and inconsistencies between quantitative and qualitative results adequately addressed?	5.5. Do the different components of the study adhere to the quality criteria of each tradition of the methods involved?	
Ericson, Hesla, and Stadskleiv (2022)^a^	Yes	Yes	No	No	Yes	No	No	1/5
Heifetz and Dyson (2017)^a^	Yes	Yes	No	No	No	No	No	1/5

^a^ Ericson, Hesla, and Stadskleiv (2022) and Heifetz and Dyson (2017) are mixed‐methods studies and therefore have been evaluated in all appropriate method‐specific sections.

## 4 Discussion

Studies involving 10 different interventions for parents of adolescents with intellectual disbility were included in this systematic review. Half of the interventions incorporated mindfulness‐based or relaxation strategies to some extent, including one of the two parenting interventions (McMahon, Wilson, and Sharry 2023), three of the four parent well‐being interventions (Hu et al. 2010; Reid et al. 2016; Singh et al. 2019, 2021) and one of the four interventions working through parents to influence adolescent outcomes (Heifetz and Dyson 2017). Mindfulness practices have previously been found to have psychological benefits for individuals with intellectual disability (Currie, McKenzie, and Noone 2019) and well‐being benefits for primary caregivers of children with an intellectual or developmental disability (Bazzano et al. 2010). Results across these interventions suggest that mindfulness‐based approaches may also be beneficial for parents of adolescents with intellectual disability in terms of mental health/well‐being (particularly stress), use of mindful parenting and parental satisfaction.

Although parenting skills were considered in five different interventions (Blankestein et al. 2019; Heifetz and Dyson 2017; McMahon, Wilson, and Sharry 2023; Reid et al. 2016; Schuiringa et al. 2017), only two interventions (McMahon, Wilson, and Sharry 2023; Schuiringa et al. 2017) predominantly focused on improving parenting skills and/or the parent–adolescent relationship. This suggests a gap in research into interventions that offer support in parenting strategies to parents of adolescents with intellectual disabilities.

Regarding intervention effectiveness, there is currently limited evidence. However, the evidence base is strongest for the parenting interventions, PPSN (McMahon, Wilson, and Sharry 2023), SST (Schuiringa et al. 2017) and the parent well‐being intervention MBPBS (Singh et al. 2021). These RCTs included well‐described interventions, scoring between 9 and 10 on the TiDieR checklist. For all three interventions, improvements were reported in adolescent behaviour. Studies of PPSN (McMahon, Wilson, and Sharry 2023) and SST (Schuiringa et al. 2017) also reported improvements in parenting practises. However, caution is needed when interpreting these results due to small and moderate effect sizes for SST and PPSN respectively. Furthermore, none of these RCTs met the full quality criteria, indicating the need for higher‐quality RCTs in this area.

In terms of parent experience, all studies offer positive social validity information; quantitative outcomes across interventions found that parents positively evaluated the intervention content in terms of helpfulness (Picard, Morin, and De Mondehare 2014), acceptability, (Blakeley‐Smith et al. 2021) or relevance (Ericson, Hesla, and Stadskleiv 2022). The three studies that incorporated a qualitative approach (Ericson, Hesla, and Stadskleiv 2022; Heifetz and Dyson 2017; Reid et al. 2016) all emphasised that parents placed value in meeting other parents, therefore, developers of future interventions might consider a group format. However, all studies that evaluated parent experience had samples of 30 parents or fewer, future research investigating the experience of parents participating in these interventions requires larger samples. Although social validity information is useful, these studies lack robust investigations into how these interventions work. To develop appropriate interventions for these families, an understanding of how and why interventions work is essential.

Previous parent well‐being interventions in the area of intellectual and developmental disabilities have reported similar improvements in parent mental health through mindfulness‐based interventions and high satisfaction ratings (Lunsky et al. 2017, 2021). Although these interventions include adolescents with intellectual disability, parents of these individuals are not specifically targeted. Interventions working through parents to influence adolescent outcomes with parents of autistic adolescents and anxiety, aiming to reduce adolescent anxiety have found promising preliminary results (Hepburn et al. 2016). Similarly, although this study included adolescents with intellectual disability, these individuals were not the primary target population.

### 4.1 Limitations

The evidence presented is limited by the lack of representation; only Hu et al. (2010) conducted a study in a country outside of Europe or North America. This may be a consequence of restricting publications to the English language; by including studies not published in English, future research may provide a more representative understanding of interventions for parents of adolescents with an intellectual disability. In addition, based on the lack of fathers participating in these interventions and fathers being consistently unrepresented in intellectual disability research (Bogossian et al. 2019), it is clear more resources are required to encourage the participation of fathers. Articles also provided limited detail on parent characteristics which would be useful to provide a clearer understanding of the type of parents participating in these interventions.

Limitations of this review include the exclusion of case studies with fewer than three participants, which risks the exclusion of certain interventions and the subsequent lack of single‐case experimental design effectiveness evidence. However, the minimum quality criterion for multiple baseline design studies requires at least three participants/replications (Kratochwill et al. 2010). Another limitation of this review is the initial moderate agreement rate of the MMAT between the two reviewers, although discrepancies were resolved through a discussion, more confidence could be held in the reliability of scores if the initial rate of agreement was higher.

### 4.2 Clinical Implications

Clinicians may consider the use of the interventions with the strongest evidence base; for parenting programmes, SST (Schuiringa et al. 2017) and PPSN (McMahon, Wilson, and Sharry 2023) may be most suitable. If the aim is to improve parental well‐being, MBPBS (Singh et al. 2021) has the strongest current evidence base. Alternatively, based on the lack of evidence and RCTs, clinicians may wish to adapt existing interventions for use with parents of adolescents with intellectual disability. Adaptations reported in these interventions may be of some use, for example, Blakeley‐Smith et al. (2021) and Singh et al. (2019) note how the intervention length was shortened to accommodate parents of adolescents with intellectual disability. Further, when adolescents were also participating in these interventions, adaptations to the intervention content were described such as accommodating to different language and cognitive abilities, and the use of visual cues and supports (Blakeley‐Smith et al. 2021; Blankestein et al. 2019; Ericson, Hesla, and Stadskleiv 2022). Notably, articles that reported group size highlighted a theme of maintaining a limited number of participants for successful intervention delivery (Blakeley‐Smith et al. 2021; Ericson, Hesla, and Stadskleiv 2022; Schuiringa et al. 2017). What is offered by clinicians needs to be driven by families' needs. For example, as previously highlighted, connecting with other parents was valued across intervention types and, therefore, an important feature for this population.

### 4.3 Further Research

It is promising that SST (Schuiringa et al. 2017) appears to be beneficial for families to some extent, PPSN may lead to improvements in multiple areas of the family (McMahon, Wilson, and Sharry 2023) and research on MBPBS (Singh et al. 2021) reports evidence of lasting improvements for families. These interventions could be prioritised for future research, including high‐quality RCTs to draw conclusions about intervention effectiveness. More focus is needed on parenting interventions that aim to improve parenting strategies and the parent–adolescent relationship. In addition, process evaluations and mediation analyses are required to develop an understanding of how these interventions work.

### 4.4 Conclusion

This review highlights the limited evidence available for supports for parents of adolescents with intellectual disabilities. There is insufficient evidence to allow for clear conclusions about the efficacy or effectiveness of existing interventions. Research on some interventions reported parents' experiences were positive but lacked exploration of how and why interventions work.

## Author Contributions

Emma Scripps completed searches and selected articles. Emma Scripps and Daniel Sutherland independently selected articles and retrieved data. Emma Scripps wrote up the manuscript with supervision from Kylie M. Gray, Richard P. Hastings and Peter E. Langdon. Kylie M. Gray, Richard P. Hastings and Peter E. Langdon also reviewed and edited the manuscript.

## Ethics Statement

The authors have nothing to report.

## Conflicts of Interest

The authors declare no conflicts of interest.

## Supporting information


**Supplementary Information S1.** PRISMA 2020 abstract checklist.

**Supplementary Information S2.** PRISMA 2020 checklist.

**Supplementary Information S3.** Full search strategy for all electronic databases.
