# Supplementary material for: Supporting Parents of Adolescents With Intellectual Disabilities: A Systematic Review of Interventions
Source: J Appl Res Intellect Disabil. 2025 Feb 19;38(1):e70004. doi: 10.1111/jar.70004 (PMC11836638; doi:10.1111/jar.70004)
Supplement: Supplementary file 3 — Supplementary Information S3. Full search strategy for all electronic databases. [file JAR-38-e70004-s003.docx]

**Supplementary Information S3:** Full search strategy for all electronic databases.

PsycINFO/MEDLINE/EMBASE:

1. ("Intellectual* Disab*" or "Intellectual* Impair*" or "Intellectual Disorder").ti,ab.
2. ("Learning Disab*").ti,ab.
3. ("Mental Handicap*" or "Mental Retard*").ti,ab.
4. ("Development* Disab*").ti,ab.
5. ("Autis*" or "Autism Spectrum Disorder*" or "ASD" or "Pervasive Developmental Disorder" or "PDD").ti,ab.
6. ("Down* syndrome" or "Fragile X syndrome" or "Smith-Magenis syndrome" or "Rett* syndrome" or "Williams syndrome" or "Lesch-Nyhan syndrome" or "Prader-Willi syndrome" or "Angelman syndrome" or "Cri*du*chat syndrome" or "Cornelia de Lange syndrome" or "Rubinstein-Taybi syndrome" or "velocardiofacial syndrome" or **"**22q11 syndrome**"** or "DiGeorge syndrome" or "Wolf-Hirschhorn syndrome" or "Kabuki syndrome" or "Fetal alcohol syndrome" or "Overgrowth syndrome").ti,ab.
7. #1 OR #2 OR #3 OR #4 OR #5 OR #6
8. (parent* or carer* or mother* or "step*mother*" or father* or "step*father*" or "foster*carer*" or caregiv* or "foster*mother*" or "foster*father*" or "foster*parent*" or maternal or paternal).ti,ab.
9. (therap* or treat* or counsel* or interven* or psychotherap* or psychoeducation*).ti,ab.
10. (adolescen* or teen* or "young adult*" or "young person" or youth or pubescent or youngster* or "young people*" or "young men" or "young women").ti,ab.
11. #8 AND #9 AND #10
12. #7 AND #11

Applied Social Sciences Index & Abstracts (ASSIA)

1. title(("Intellectual* Disab*" or "Intellectual* Impair*" or “Intellectual Disorder”) OR (“Learning Disab*”) OR ("Mental Handicap*" or "Mental Retard*") OR ("Development* Disab*") OR ("Autis*" OR "Autism Spectrum Disorder*" OR "ASD" OR "Pervasive Developmental Disorder" OR "PDD")) OR abstract(("Intellectual* Disab*" or "Intellectual* Impair*" or “Intellectual Disorder”) OR (“Learning Disab*”) OR ("Mental Handicap*" or "Mental Retard*") OR ("Development* Disab*") OR ("Autis*" OR "Autism Spectrum Disorder*" OR "ASD" OR "Pervasive Developmental Disorder" OR "PDD"))
2. title(("Down* syndrome" OR "Fragile X syndrome" OR "Smith-Magenis syndrome" OR "Rett* syndrome" OR "Williams syndrome" OR "Lesch-Nyhan syndrome" OR "Prader-Willi syndrome" OR "Angelman syndrome" OR "Cri*du*chat syndrome" OR "Cornelia de Lange syndrome" OR "Rubinstein-Taybi syndrome" OR "velocardiofacial syndrome" OR **"**22q11 syndrome**"** OR "DiGeorge syndrome" OR "Wolf-Hirschhorn syndrome" OR "Kabuki syndrome" OR "Fetal alcohol syndrome" OR "Overgrowth syndrome")) OR abstract(("Smith-Magenis syndrome" OR "Rett* syndrome" OR "Williams syndrome" OR "Lesch-Nyhan syndrome" OR "Prader-Willi syndrome" OR "Angelman syndrome" OR "Cri*du*chat syndrome" OR "Cornelia de Lange syndrome" OR "Rubinstein-Taybi syndrome" OR "velocardiofacial syndrome" OR **"**22q11 syndrome**"** OR "DiGeorge syndrome" OR "Wolf-Hirschhorn syndrome" OR "Kabuki syndrome" OR "Fetal alcohol syndrome" OR "Overgrowth syndrome"))
3. 1 OR 2
4. title((parent* OR carer* OR mother* OR “step*mother*” OR father* OR “step*father*” OR “foster*carer*” OR caregiv* OR “foster*mother*” OR “foster*father*” OR “foster*parent*” OR maternal OR paternal)) OR abstract((parent* OR carer* OR mother* OR “step*mother*” OR father* OR “step*father*” OR “foster*carer*” OR caregiv* OR “foster*mother*” OR “foster*father*” OR “foster*parent*” OR maternal OR paternal)))
5. title((therap* OR treat* OR counsel* OR interven* OR psychotherap* OR psychoeducation*)) OR abstract((therap* OR treat* OR counsel* OR interven* OR psychotherap* OR psychoeducation*))
6. title((adolescen* OR teen* OR "young adult*" OR "young person" OR youth OR pubescent OR youngster* OR "young people” OR "young men" OR "young women")) OR abstract((adolescen* OR teen* OR "young adult*" OR "young person" OR youth OR pubescent OR youngster* OR "young people” OR "young men" OR "young women"))
7. #3 AND #4 AND #5 AND #6

Web of Science:

1. (TI=("Intellectual* Disab*" or "Intellectual* Impair*" or “Intellectual Disorder”) OR (AB=("Intellectual* Disab*" or "Intellectual* Impair*" or “Intellectual Disorder”)))
2. (TI=(“Learning Disab*” ) OR (AB=(“Learning Disab*”)))
3. (TI=("Mental* Handicap*" or "Mental* Retard*") OR (AB=("Mental* Handicap*" or "Mental* Retard*")))
4. (TI=("Development* Disab*") OR (AB=("Development* Disab*")))
5. (TI=("Autis*" or "Autism Spectrum Disorder" or "ASD" or "Pervasive Developmental Disorder" or "PDD") OR (AB=("Autis*" or "Autism Spectrum Disorder" or "ASD" or "Pervasive Developmental Disorder" or "PDD”)))
6. **(TI=(**"Down* syndrome" or "Fragile X syndrome" or **"Smith-Magenis syndrome" or "Rett* syndrome" or “Williams syndrome” or "Lesch-Nyhan syndrome" or "Prader-Willi syndrome" or "Angelman syndrome" or "Cri*du*chat syndrome" or "Cornelia de Lange syndrome" or "Rubinstein-Taybi syndrome" or "velocardiofacial syndrome" or "**22q11 syndrome**"** or **"DiGeorge syndrome" or “Wolf-Hirschhorn syndrome” or “Kabuki syndrome” or "Fetal alcohol syndrome" or "Overgrowth syndrome") OR (AB=("**Down* syndrome" or "Fragile X syndrome" or **"Smith-Magenis syndrome" or "Rett* syndrome" or “Williams syndrome” or "Lesch-Nyhan syndrome" or "Prader-Willi syndrome" or "Angelman syndrome" or "Cri*du*chat syndrome" or "Cornelia de Lange syndrome" or "Rubinstein-Taybi syndrome" or "velocardiofacial syndrome" or "**22q11 syndrome**"** or **"DiGeorge syndrome" or “Wolf-Hirschhorn syndrome” or “Kabuki syndrome” or "Fetal alcohol syndrome" or "Overgrowth syndrome")))**
7. #1 OR #2 OR #3 OR #4 OR #5 OR #6
8. (TI=(parent* OR carer* OR mother* OR “step*mother*” OR father* OR “step*father*” OR “foster*carer*” OR caregiv* OR “foster*mother*” OR “foster*father*” OR “foster*parent*” OR maternal OR paternal) OR (AB=(parent* OR carer* OR mother* OR “step*mother*” OR father* OR “step*father*” OR “foster*carer*” OR caregiv* OR “foster*mother*” OR “foster*father*” OR “foster*parent*” OR maternal OR paternal)))
9. (TI=(therap* OR treat* OR counsel* OR interven* OR psychotherap* OR psychoeducation*) OR (AB=(therap* OR treat* OR counsel* OR interven* OR psychotherap* OR psychoeducation*)))
10. (TI=(adolescen* OR teen* OR “young adult*” OR “young person” OR youth OR pubescent OR youngster* OR “young people*” OR “young men” OR “young women”) OR (AB=(adolescen* OR teen* OR “young adult*” OR “young person” OR youth OR juvenile OR pubescent OR youngster* OR “young people*” OR “young men” OR “young women”)))
11. #8 AND #9 AND #10
12. #7 AND #11
